# Modeling an Edge Computing Arithmetic Framework for IoT Environments

**DOI:** 10.3390/s22031084

**Published:** 2022-01-30

**Authors:** Pedro Juan Roig, Salvador Alcaraz, Katja Gilly, Cristina Bernad, Carlos Juiz

**Affiliations:** 1 Computer Engineering Department, Miguel Hernández University, 03202 Elche, Spain; salcaraz@umh.es (S.A.); cbernad@umh.es (C.B.); 2 Mathematics and Computer Science Department, University of the Balearic Islands, 07022 Palma de Mallorca, Spain; cjuiz@uib.es

**Keywords:** edge computing, fog computing, CNN, formal modeling, ACP, Promela, Spin

## Abstract

IoT environments are forecasted to grow exponentially in the coming years thanks to the recent advances in both edge computing and artificial intelligence. In this paper, a model of remote computing scheme is presented, where three layers of computing nodes are put in place in order to optimize the computing and forwarding tasks. In this sense, a generic layout has been designed so as to easily achieve communications among the diverse layers by means of simple arithmetic operations, which may result in saving resources in all nodes involved. Traffic forwarding is undertaken by means of forwarding tables within network devices, which need to be searched upon in order to find the proper destination, and that process may be resource-consuming as the number of entries in such tables grow. However, the arithmetic framework proposed may speed up the traffic forwarding decisions as relaying on integer divisions and modular arithmetic, which may result more straightforward. Furthermore, two diverse approaches have been proposed to formally describe such a design by means of coding with Spin/Promela, or otherwise, by using an algebraic approach with Algebra of Communicating Processes (ACP), resulting in a explosion state for the former and a specified and verified model in the latter.

## 1. Introduction

The development of IoT (Internet of Things) technologies are sharply rising in recent times, thanks to the advances in AI (Artificial Intelligence) and its application to MEC (Multi-Access Edge Computing) environments [1]. This union of both concepts is labelled as Edge AI [2], which brings about powerful data centres to carry out many complex computing tasks in servers located around the edge of the network, as opposed to in those situated up in the cloud premises, thus enhancing performance [3].

The Edge AI paradigm is critical to undertake the processing of all big data generated for the applications being run by the ever growing amount of IoT devices [4], hence allowing these to obtain responses with much lower latency and a far smaller amount of bandwidth compared to those being obtained if cloud servers were used [5].

This way, computational intelligence in IoT deployments is basically located on the edge devices, those being either end devices or edge servers [6], as usually IoT devices have constraint resources, which makes them outsource most computing tasks to the edge [7]. Furthermore, some of those tasks might also be offloaded up to the fog servers (provided there is a fog deployment up and running), or otherwise, up to the cloud servers (which are supposed to be always on) [8].

Additionally, the remote computing layer where a given server is located, that being either at the edge layer, at the fog tier or at the cloud facilities, involves having more computing and storage power as it gets closer to the cloud, which implies having more analytic power related to AI operations. This way, as end devices are hanging on edge servers, whereas those are connected to fog servers, and these are linked to cloud, it results in fog servers having more capabilities than edge ones, whereas cloud ones being the most proficient regarding remote computing capacities [9].

Edge AI may represent a huge advance in many business fields, such as security and surveillance, real-time video processing, content generation or visual inspection [10]. For instance, AI-powered low-code and no-code development platforms are on the rise [11], both consisting in automatic code generation through visual flow charts, even though the former needs some small amount of hand-written code, whilst the latter does not need any [12]. It is forecast that by 2024, up to 65% of the overall application development will use any of both approaches, thus facilitating content generation [13].

Other examples of Edge AI implementations are dedicated to basically any sort of activity, those being as disjointed as such as smart office automation [14], exoskeleton manipulation [15], wearable devices [16] or screening for myocardial infarctions [17].

In this paper, a formal model for a generic remote computing deployment scenario is going us to be exposed and proposed in a coding fashion, by means of a modeling language called Protocol/Process Meta Language (Promela) [18] and a model checker named Spin [19], and then, that generic scenario is also going to be presented and exhibited by means of an abstract process algebra called Algebra of Communicating Processes (ACP) [20].

The reason for choosing both Formal Description Techniques (FDT) to construct models is to represent the same system according to two different approaches. With respect to Spin/Promela, it is a time-based software simulation, which is considered as a timed FDT, thus allowing us to focus on quantitative characteristics of the system being modeled [21]. With regards to ACP, it does not contain any time implications, so it is branded as a timeless FDT, thus permitting us to focus on qualitative characteristics of the modeled system [22].

Generally speaking, FDT are mainly focused on studying distributed systems being run in a non deterministic fashion so as to check for abnormal conditions, leading to malfunction and degradation in system performance, thus posing a risk to system integrity. The most popular of those conditions is deadlock, which may be provoked by different circumstances such as mutual exclusion or circular wait, whereas other conditions may arise, such as livelock, data race or resource starvation [23].

It is to be noted that this paper presents a theoretical study to build up an arithmetic-based framework aimed at edge computing wired designs in order to optimize packet forwarding by just applying integer divisions and modular arithmetic [24], and that approach will be put into practice by means of a Spin/Promela design and an ACP model presented in an incremental way. On the other hand, packet forwarding in real scenarios is usually undertaken by traditional routing and switching, which need to search the next hop towards the expected destination through looking up into forwarding tables, such as routing tables for internetwork communications or mac-address tables for intranetwork communications, the former being at layer 3 and the latter being at layer 2 within the OSI conceptual model for network communications.

However, the arithmetic framework proposed herein does not need any forwarding table whatsoever, but it only needs to apply the proper arithmetic operations involving the source host identifier, namely *a*, the destination host identifier, namely *b*, and the value of parameter *k*, in a similar way as it happens in a fat tree architecture [25]. Therefore, the advantage of this framework may be about getting an alternative way to packet forwarding when dealing with either routing or switching, as this framework avoids the time spent looking at routing or forwarding tables, and substitute those searches by simple arithmetic operations.

The model proposed is made up according to a layered approach, where different scenarios have been proposed by increasing the number of tiers being under consideration, starting small and growing until the whole model is constructed. The layers involved are cloud, fog, edge and end devices, even though a lower layer composed by sensors and actuators might be also added by following the same pattern of action.

In that context, the whole framework depends on a generic parameter *k*, by means of applying a hub and spoke topology at all layers, where any item located at a particular layer acts as a one hub and gets connected to *k* items located at the directly connected lower layer, which act as spokes. The same structure gets repeated at all layers, whereas a full mesh topology is further imposed at fog layer for operability purposes.

Therefore, this structure composed of hierarchical tiers with the aforesaid interconnections makes possible the advantages to the framework proposed due to the optimal paths offered when applying integer divisions and modular arithmetic. However, the number of items within each layer must exactly match the necessary figures depending on parameter *k*, or otherwise, suboptimal paths may arise as integer divisions and modular arithmetic would not work out properly. Hence, the strict shape of such a structure may also be responsible of its drawbacks, provoking that forwarding tables will achieve better results if some item at any layer is missing.

Regarding performance evaluation in the proposed scenario, it is to be said that, in theory, working with integer divisions to find out the next hop and dealing with modular arithmetic to discover the port involved to get there appears to be easier than searching through the different number of entries making up a forwarding table, which might theoretically account for faster processing times in routing and switching.

Nonetheless, from a practical point of view, the real gain in performance when dealing with the traffic forwarding obtained when applying the arithmetic framework proposed as opposed to searching within the proper forwarding tables actually will depend on the time being spent in making the appropriate calculations on real gear, as opposed to the time being used in carrying out the search in the appropriate tables, even though it is to be noted that the former beats the latter regarding theory calculations because integer divisions are dealt with faster than searching through a table, so the same result is expected to happen when putting them into practice.

In both cases, the hardware and software features of the computing assets being used to undertake either the arithmetic operations or the searches will indicate which one does it more efficiently, which will depend on how the operations are implemented in the ASIC hardware and the software complements being in charge of performing those tasks, as this point may render one approach more efficient than the other.

Regardless, the goals in this paper are to first review the theory behind the arithmetic framework proposed for edge computing environments, where IoT current trends are outlined. At that point, the fundamentals of the framework proposed are described, which is followed by the presentation of a model coded in Promela and executed in Spin in order to check its feasibility. Afterwards, some models coded in ACP are presented in an incremental manner so as to study its suitability.

The organization of the remaining of this paper goes as follows: Section 2 presents the IoT current trends; afterwards, Section 3 introduces the basics of the design considered; next, Section 4 delivers a model with Spin/Promela; after this, Section 5 proposes a model with ACP; and finally, Section 6 draws some final conclusions.

## 2. Iot Current Trends

IoT (Internet of Things) is already a reality at this time and age, where the disruption due to Covid-19 pandemic worked as a catalyst for digital transformation in all sectors [26]. Actually, the IoT deployments are growing massively, offering new services and scenarios [27]. Furthermore, the introduction of 5G cellular communications will open up new opportunities related to IoT in virtually any field of application [28].

IoT industry is forecast to grow at a double digit rate in the coming years [29], whilst the amount of connected devices will rise to 46 billion by the end of 2021, with a market value of over USD 500 billion [30]. Among all their possible uses, the main IoT trends for business in 2021 and beyond are the following [31]:

- AIoT (Artificial Intelligence of Things): AIoT analytics boost functionality of heterogeneous IoT devices by combining information and knowledge [32].
- Edge computing: Edge AI is a key player in computing efficiency as it brings cloud AI capabilities straight to devices distributed at the edge of the network [33].
- Customized experience: A great deal of IoT success depends on the degree of personalized services being offered in order to match client expectations [34].
- Connectivity: IoT communications may employ a wide range of technologies to connect to edge servers, such as 5G, WiFi 6, Bluetooth or Zigbee [35].
- Smart cities: IoT technologies help implement loads of opportunities in all areas of a city so as to improve the lives of their citizens [36].
- IoT-based healthcare: Disease diagnostics and health monitoring has been transformed from hospital-centric to patient-centric thanks to IoT devices [37].
- VANET (Vehicular Ad-Hoc Networks): Autonomous Vehicles (AV) make use of some sensors and software to check the environmental conditions and drive without human intervention [38].
- IIoT (Industrial Internet of Things): Edge computing and IoT allow a fully connected, automated and intelligent environment to improve efficiency applied to industrial processes [39].
- G-IoT (Green IoT): Edge AI deployments lead to higher energy consumption; hence, the new G-IoT paradigm is aimed at reducing the overall carbon footprint [40].
- IoT serverless: Serverless edge computing allows the execution of on-demand code related to IoT applications throughout the edge infrastructure [41].
- IoFT (Internet of Federated Things): AI training is usually carried out on the cloud premises (CL), although many IoT applications require to do it right on the edge servers (FL) [42], leading to a paradigm where devices may extract knowledge collaboratively to build analytic models whilst keeping privacy [43].
- Energy efficiency: Energy harvesting techniques may supply power to IoT devices from a suitable source of ambient energy, such as solar, thermal, wind, vibrations or radio frequency [44].

Apart from all those hot topics within IoT, it is worth mentioning that the first edge computing deployments were focused on data collection and application delivery, although they have now evolved into data processing, which require higher performance and scalability. Basically, the key point of a good edge computing strategy is to reduce latency when response times are critical to increase performance and safety, as business success or fail depending on the insights they may get out of data and how fast they may do it [45].

Some of the key factors when dealing with an edge computing strategy are system integration platforms, where information and operation technologies converge, ecosystems and alliances, where open platforms are ever more important, trust and trustworthiness, where digital trust leverages the digital transformation, hyperconverged infrastructure, where the use of commodity hardware being customized by software brings computing systems to a new paradigm, and visionary concepts, such as swarm computing and decentralized self-organized systems [46].

## 3. Computational Intelligence and Iot

One of the main features related to IoT devices is the constraint capabilities regarding processing power, memory, storage, bandwidth and power supply. Those restrictions when dealing with its own resources lead to move most computing tasks to remote locations. This way, IoT devices may merely acquire external information from the environment through some sensors and forward such raw data on to a particular edge device for it to undertake the appropriate processing, which involves the encapsulation of such data in the proper way according to the communication protocol being used to get in touch with the corresponding edge device [47].

On the other hand, when the aforesaid edge device brings processed data back in response to a former request, it first decapsulates such data so as to extract the relevant information, and in turn, send them to the appropriate actuator, which may merely act on the environment according to such information received [48].

From the point of view of networking, sensors and actuators may be seen as endpoints, whose traffic flows are sent over to the default gateway in the former, or the other way around in the latter, where such a default gateway may account for a given edge device. Moreover, it is irrelevant for a sensor which device is going to do the processing up in the remote computing hierarchy, as long as the related responses come back to the proper actuator in a reasonable time, hence no matter whether the server dealing with a given request is located on the edge, on the fog or on the cloud [49].

Regarding the remote computing levels considered in this paper, edge nodes may be seen as connected to IoT devices, thus being the lower layer to take part in remote computing. Then, fog nodes may be denoted as the intermediate layer, as they interconnect edge nodes. In turn, cloud nodes may be described as the upper layer, as they interconnect fog nodes. In view of that, I may seem clear that fog nodes ought to be more powerful than edge ones and cloud ones should usually be the highest.

## 4. Basic Model Proposed

The foundation of the design proposed for a framework of remote computing system is composed by two processing levels, such as an edge layer, where end devices get connected, although sometimes IoT devices might also do, and a fog layer, where edge layers are connected, taking into account that all fogs are interconnected by a full mesh topology, thus being all just one hop away to the rest of them.

Furthermore, a cloud layer might be also included, that being connected to all fog nodes forming a hub and spoke topology, even though no cloud level is actually being incorporated to this fundamental model so as to keep it simple, as cloud might only take part as a backup entity for processing, storage or offloading, and that might distract from the relevant traffic flows to go from a source end to destination end. At a later stage, a cloud layer will be included as an enhancement of such a model.

Regardless, regarding the layout of all available layers involved in the framework proposed and the interconnections among them, nodes staying in the edge layer communicate with end devices by means of their downlink ports and with fog nodes by means of their uplink ports, whereas nodes located in the fog layer communicate with edge nodes by means of their downlink ports and with both the rest of fog nodes along with the cloud node (only if there is a cloud facility available in the model) by means of their uplinks ports.

The essential part of this design is going to be modeled according to basic arithmetic operations, where integer division and modular arithmetic play a capital role. In this sense, the former yields the integer quotient of a regular division, also known as floor function, that being represented by $\left\lfloor a\;/\;k\right\rfloor$, whereas the latter plays with the set of its possible remainders, that being denoted by $a_{|k}$. Furthermore, the integer division in excess, also known as ceil function, is also used herein, which is described by $\left\lceil a\;/\;k\right\rceil$.

**Figure 1:**
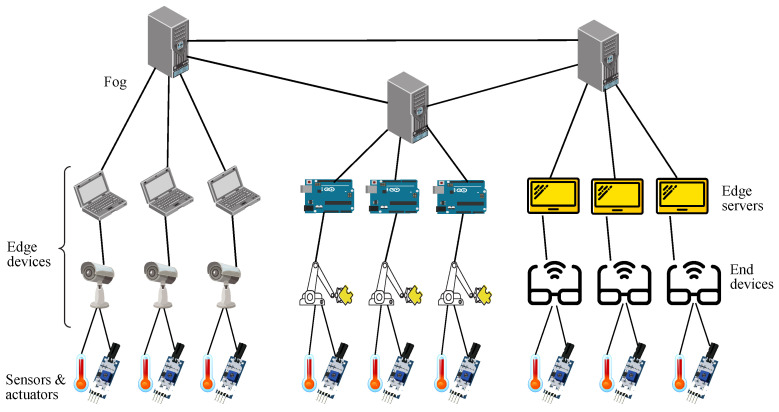
Deployment of a basic model with 3 fog nodes.

With that in mind, the design proposed consists of a group of *k* fog servers being interconnected, where each of those has a bunch of *k* edge servers connected to them, whilst each of them has a group of *k* end devices with IoT items linked to them. However, for simplicity purposes, it is going to be defined a basic model where each edge node has only one end device attached to it, where each of those just has one sensor and one actuator connected. This way, the end device identifier also indicates its attached sensor and its attached actuator, which may facilitate to understand how this basic model works by just applying the arithmetic operations cited above. Therefore, the overall picture described for the basic model may be appreciated in Figure 1 for a value k=3, with *k* fog nodes, *k* edge nodes per fog node, 1 end device per edge node, and 1 sensor and 1 actuator per end device.

In this context, the key point in that basic layout is the value of variable *k*, such that there are just *k* fogs being interconnected according to a full mesh topology, whilst there are just *k* edges hanging per each fog. Hence, every fog has a direct link to all other fogs, which allows for the shortest paths among them. This infrastructure results in three different types of communication, such as intraedge for end devices connected to the same edge, intrafog for end devices connected to the same fog, and interfog for end devices connected to different fogs.

However, thanks to this basic model having just one sensor and one actuator per end device, which is unique for each edge, then intraedge communications are obvious because there are only one uplink channel from a sensor towards an end device and one downlink channel from an end device to an actuator, whilst having a unique link from each end device to its associated edge node, hence all those communications are omitted in the basic model so as to attain further simplification.

Regardless, considering that sensors and actuators are attached to their respective end devices, it happens that intraedge communications may always contain 2 links, such as the one to go from the source end device, where a transmitting sensor is connected, to the common edge, and the one to go from there to the destination end device, where a receiving actuator is connected to.

In this sense, this basic model also exhibits intrafog communications, which always consist of 4 links, such as from source end device to source edge, from there to their common fog node, from there to destination edge, and from there to destination end device. Furthermore, this basic model exposes interfog communications as well, which always require 5 links, such as from source end device to source edge, from there to source fog, from there to destination fog, from there to destination edge, and from there to destination end device.

**Figure 2:**
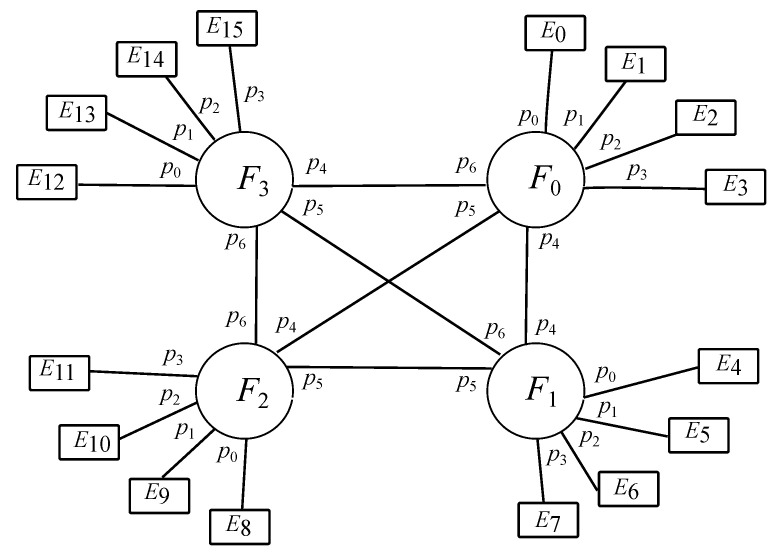
Full mesh layout for *k* = 4.

Figure 2 exhibits an instance of the design where k=4. Nonetheless, no matter the value of *k*, the amount of links for each type of communication is represented by the numbers given above. It is to be noted that different interfog topologies from full mesh would account for a larger number of links when dealing with interfog communications.

From that picture, it seems clear that fog items have been numerated from 0 to k−1 clockwise, as well as edge items have been done from 0 to $k^{2}-1$ clockwise, although each category gets identified independently. It is to be noted that the identifiers of all edges hanging on the same fog match their outcome when applying the integer division of such an identifier by *k*, which at the same time happens to match the fog identifier where they are all being connected to.

With regards to the port numbers, edge devices account for a unique uplink port, which is not being cited for simplification purposes. Furthermore, the unique downlink port on each edge device is not being considered either for the same reason, which also applies to the sensor and the actuator hanging on each end device. On the contrary, port numbers for fog devices are indeed cited, such as downlink ports going towards its connected edges are labeled from 0 to k−1, all of them being congruent modulo *k* with the corresponding edge identifiers, such that this arithmetic operation is used to obtain the port to the edge destination. Likewise, uplink ports going towards the rest of the fogs are marked from *k* to 2k−2, all those being assigned in an increasing order related to the ascending order of the other fog identifiers.

This sort of numerical arrangement to identify both the edges and fogs, as well as their ports, permit to construct a model to express the behavior of such a system by employing only arithmetic operations, which may allow to obtain the string of devices and their ports forming the path between a given source edge towards a given destination edge.

Furthermore, as the basic model only considers one end device per edge, it occurs that a source edge identifier also accounts for the source end device, whereas a destination edge identifier also does for the destination end device, because only one end device is considered per edge. Likewise, as the basic model considers one single sensor and one sole actuator per end device, it also happens that any edge identifier accounts not only for its connected end device, but also for the sensor and the actuator hanging on it. Therefore, the basic model only needs to focus on traffic flows among edges and fogs, whilst considering the ports involved in the latter.

Focusing on the model, each communication just quotes two parameters in the form of natural numbers within the range of $Z_{k^{2}}=\left\{0\cdots k^{2}-1\right\}$, such as the source edge (where the source end device is hanging on, which contains the active sensor, that is, the one transmitting data) and the destination edge (where the destination end device is connected to, which includes the active actuator, that is, the one receiving data). With this in mind, the integer division of those by *k* may allow to obtain the source fog and the destination fog, respectively, which is a natural number within the range of $Z_{k}=\left\{0\cdots k-1\right\}$. Once the source and destination fogs have been found out, there is always a link between them both, as the network topology among fogs is full mesh; hence, no other fogs may be taken into consideration to attain all devices involved between a source and a destination edge.

With respect to the ports involved, those in the edges are irrelavant in this basic model, as there is just one per edge. On the contrary, each fog has *k* downlink ports, thus looking at its *k* connected edges, and k−1 uplink ports, hence going to each one of the other k−1 fogs. The downlink ports are identified sequentially within the interval {0⋯k−1}, where the edge identifier hanging on each port is congruent modulo *k* with the port identifier. On the other hand, the uplink ports are identified according to the interval {k⋯2(k−1)}, each one pointing to the rest of the fogs in ascending order.

In this setup for uplink ports between a source fog *i* and a destination fog *j*, the source end *p* of each link may be calculated through Equation (1), where the destination end *q* of such a link may be found out with Equation (2).

(1) $$\mathrm{Source}\;\mathrm{Port}\left(p\right)=\left\{\begin{array}{c} \mathrm{if}\;i<j\;⟶\;k+j-1\\ \mathrm{if}\;i>j\;⟶\;k+j \end{array}\right.$$

(2) $$\mathrm{Destination}\;\mathrm{Port}\left(q\right)=\left\{\begin{array}{c} \mathrm{if}\;i<j\;⟶\;k+i\\ \mathrm{if}\;i>j\;⟶\;k+i-1 \end{array}\right.$$

Additionally, Equation (1) may be collapsed into just one arithmetic Equation (3), which aggregates both cases by adding up a specific coefficient whose value is either one if i<j, or otherwise, it is zero if i>j. Such a coefficient is found out by first calculating the integer division of *j* by i+1 (thus resulting in a value greater than 0 if i<j, or just 0 if i>j, whilst adding up 1 to the divisor eliminates the risk of dividing by zero), and then applying the integer division in excess by *k* (thus resulting in 1 if i<j, or 0 if i>j). Likewise, Equation (2) may also be collapsed into only one arithmetic Equation (4) following the same reasoning, but swapping *i* for *j*.

(3) $$\mathrm{Source}\;\mathrm{Port}\left(p\right)=k+j-\left\lceil \frac{\left\lfloor \frac{j}{i+1}\right\rfloor }{k}\right\rceil$$

(4) $$\mathrm{Destination}\;\mathrm{Port}\left(q\right)=k+i-\left\lceil \frac{\left\lfloor \frac{i}{j+1}\right\rfloor }{k}\right\rceil$$

As an example, Table 1 exposes the interfog links in a unidirectional way among all fog nodes for the particular case where k=4. In this case, as there are 4 fog nodes, the establishment of full connectivity among them all induces k×(k−1)/2 links, which results in 6 bidirectional channels for k=4, thus accounting for 6×2=12 unidirectional channels.

Considering a generic *k* value, it is to be noted that each single unidirectional interfog channel may be represented by a unique combination of a source fog, a destination fog and the link between them, that being described by both its source end and its destination end. In this context, all available unidirectional interfog channels may be mapped to an array in order to facilitate the coding implementation of a Promela model by associating each of those with an sole identifier. In that case, variable *m* may be an array of natural numbers, where obviously its indexes match the corresponding values being stored on them, and whose target is to identify each one of the aforesaid unique combinations, thus acting as a mapping array between each given instance of unidirectional interfog channel to a particular natural number.

In this sense, on the one hand, Equation (5) finds the mapping of the source end for all unidirectional interfog channels, where each particular instance is associated to a unique pair composed by a given source fog and one of its uplink ports. On the other hand, Equation (6) states the mapping of all destination ends for the aforementioned channels, where each given instance is bounded to a sole pair consisting of a particular destination fog and one of its uplink ports.

Therefore, the mapping of a given unidimensional interfog channel may be independently calculated from the point of view of either the sender end or the receiver end. This way, Equation (5) finds the index of mapping array *m* as a function of source fog *i* and source port *p*, whereas Equation (6) does it by means of destination fog *j* and destination port *q*. Hence, the same value of *m* associated to a particular unidirectional channel is obtained with either the source fog node and its source uplink port or the destination fog node and its destination uplink port.

(5) SourcePortMapping(m)=(k−1)×i+p−k

(6) $$\mathrm{Destination}\;\mathrm{Port}\;\mathrm{Mapping}\left(m\right)={\left(\left(j-1\right)-q+k\right)}_{|k}+\left(q-k\right)\times k$$

Taking that all into account, the layout proposed simplifies a great deal all forwarding operations to be undertaken when moving traffic flows from one edge to another, as integer divisions and arithmetic modulo *k* provides all intermediate devices (those being the fog or fogs being traversed), along with their ports involved.

However, the model may be further expanded in different ways, such as considering that *k* end devices are connected to each edge in a hub and spoke fashion, which may lead to an overall amount of $k^{3}$ end devices, being numerically identified within the interval $\left\{0\cdots k^{3}-1\right\}$. That way, there may be *k* end devices hanging on each edge, where the integer division between each end device and *k* results in the edge identifier, whose downlink ports are congruent modulo *k* with their connected end devices. Analogously, there may be $k^{2}$ end devices having the same fog two hops away, which may be identified by means of the integer division between an end device and $k^{2}$, whose downlink ports are congruent modulo $k^{2}$ with those end devices.

Furthermore, cloud facilities may be included to work as a backup of the aforementioned model, where each fog may have links to those facilities. This way, a cloud server may act as backup for storage or offloading purposes, even though the processing tasks will be preferably undertaken in the lower levels.

## 5. Modeling with Spin/Promela

To start with, Promela is the acronym of PROtocol MEta LAnguage, where the first word may also be substituted with PROcess. It is a high-level specification language, as it portrays a strong degree of abstraction. Promela’s syntax is C-like, even though the latter is a programming language, whereas the former is a modeling one whose target is modeling the interactions among no-deterministic distributed systems [50]. Furthermore, such model specifications may feed Simple Promela INterpreter (SPIN) model checker in order to verify the model [51], as well as check specific properties related to Linear Temporal Logic (LTL) [52].

In this context, the basic model exposed in the previous section is going to be modelled by means of Promela code, which will further feed Spin model checker so as to verify the code and obtain some parameters out of such a code. It is to be reminded that the design of the basic model heavily depends on variable *k*, as it only takes into account *k* fog nodes, $k^{2}$ edge nodes and the same number of end devices, taking into account all the interconnection ports among them exposed in the previous chapter.

Additionally, it is to be remarked that the key arithmetic tools in this Promela model presented herein are integer division and modular arithmetic. Regarding the former, it is the default kind of division when dealing with byte and integer types in Promela, thus a simple division applies herein as the type of destination identifier is a byte; hence, calculating the associated fog node of a destination device is as straightforward as performing a division of such a destination by *k*. With respect to the latter, Promela does not have it implemented, so it has to be defined within the code by adapting the remainder theorem, hence calculating the downlink port from a fog to an edge towards destination makes use of that mathematic tool.

Furthermore, interfog communications are implemented through the establishment of a mapping array of channels which identifies each single unidirectional interfog link, as stated in the previous section. This way, all those interfog paths are perfectly distinguished, thus making it possible to clearly spot each of their sending and receiving ends by means of the aforementioned Equations (5) and (6). On the other hand, those equations require to find the source and destination ports, where Equation (1) has been employed to find the former, whereas the latter have been considered as a range of the available values, as each concrete value is irrelevant in the code proposed. Alternatively, the former might have been found by means of (3), even though the integer division in excess is not defined in Promela, so it would have been necessary to first define it.

Regarding the code for the basic model proposed, it is shown in Algorithm 1, where the first line specifies the value of variable *K*, which will be a constant value throughout the whole code snippet, whilst the second line builds up the function mod(a,n) as Promela does not have any to represent arithmetic modulo *n* [53]. Then, the third line states an abstract message type, which bears a source and a destination identifiers, whereas lines from 4 to 8 declare all message channels involved. Each of those are arrays of a bunch of channels that can store just 1 message containing its type, along with its source identifier and its destination identifier.

After that, the declarations for the three kinds of entities taking part in this model are carried out, such as Devices, Edge and Fog. It is to be said that in every channel, the nomenclature for sending a message through a channel is identified by channel ! message, whereas that for receiving a message through a channel is done by channel ? message, where the former spots the sending end and the latter does the receiving end. It is also to be noted that *i* stands for identifying both an entity and the ends of each channel where it is involved. Besides, the source and destination of each message passing through any remote computing entity, namely Edge and Fog, are Devices identified as *s* and *d*, respectively, whilst those generating a message portray *i* as a source, whereas those receiving a message carry *i* as destination.

In this sense, each Device entity generates messages where the source is its own identifier *i* and the destination is non-deterministic, which depart from itself for being the source device and go towards its directly connected edge, which in this case plays the role of source edge, through its channel from Sensor. On the other hand, each device also listens to receive messages from its channel to Actuator, those coming from its directly connected edge, which in this case plays the role of destination edge, and going towards itself for being the destination device.

Moreover, for each Edge entity, if a message is coming from a device through its channel fromSensor, then it is checked whether the current edge instance is the destination edge, and if this is the case, the incoming message is sent towards its directly connected device, which happens to be the destination device, through its channel toActuator, or otherwise, that message is forwarded on towards its directly connected fog, which occurs to be the source fog, through channel Edge2Fog. Likewise, if a message is coming from its directly connected fog, which play the part of destination fog, through its channel Fog2Edge, then it is sent towards its directly connected device, which play the role of destination device, through its channel toActuator.

Furthermore, for each Fog entity, if a message is received from an edge through its channel Edge2Fog, then it checks whether the current fog instance is the destination fog, and if that is the case, the incoming message is forwarded on towards the appropriate destination edge through its channel Fog2Edge, or otherwise, such a message is sent towards the destination fog through channel Fog2Fog, which is directly connected to the current fog thanks to the full mesh network topology among fog nodes. Likewise, if a message is received from another fog, which occurs to be the source fog, through its channel Fog2Fog, it is in turn forwarded towards the appropriate destination edge, which happens to be the destination edge, through its channel Fog2Edge.

Eventually, all instances of each type are created by means of in the init process by means of the run function, which starts up a new process for each of those instances, which will interact with each other in a non-deterministic fashion, resulting in a different outcome for each time the simulation is run.

At this stage, this basic model coded in Promela is going to be run with Spin several times by trying incremental values of parameter *k* so as to study the results produced [54]. In this sense, Table 2 presents the results for a particular execution for each of those values of k={2,3,4}, presenting the length of the state vector in bytes, the depth reached, the states stored and the states matched, that meaning the number of different states found during the simulation time.

The values obtained regarding the number of states is extremely high, as there are over $10^{7}$ for k=2 and over $10^{8}$ for k=3 and k=4. This clearly exhibits an explosion of states happening with the lowest values of *k*, which will get much higher if greater values of *k* are assigned, thus making the model unmanageable.

It is to be mentioned that the state explosion occurs when the number of states to be modeled increases with the addition of further aspects to the model, which results in an rapid increasing number of states to be included, thus leading to a fast growing number of transitions among those states, hence making the model cumbersome [55].

Therefore, the study of this basic model with Spin/Promela is discouraging due to that condition, because small values of *k* already brings results being hard to deal with, which also obviously puts off any extension of this basic model. In this situation, it is convenient to leave Spin/Promela modeling aside, which might be considered as a timed FDT, and adopt an ACP based approach to undertake the models required, which may be seen as a timeless FDT. This way, the focus will be swapped from quantitative features to qualitative ones, hence concentrating just on the relationships among entities in a non-deterministic distributed environment.
**   Algorithm 1:** Basic model coded in Promela.1 #define K 42 #define mod(a,n)   ((((a+n) % n) + n) % n)3 mtype = {MSG}4 chan fromSensor[K*K]   = [1] of {mtype, byte, byte}5 chan toActuator[K*K]    = [1] of {mtype, byte, byte}6 chan Fog2Edge[K*K]    = [1] of {mtype, byte, byte}7 chan Edge2Fog[K*K]    = [1] of {mtype, byte, byte}8 chan Fog2Fog[K*(K-1)]   = [1] of {mtype, byte, byte}9 proctype Devices (byte i) {10    byte s, d, n = 0;11    do12    :: atomic {n<1 -> select(d : 0 .. (K*(K-1)));13                       fromSensor[i] ! MSG(i,d); n++}14    :: toActuator[i] ? MSG(s,i)15    od16 }17 proctype Edge (byte i) {18    byte s, d;19    do20    :: if21      :: atomic { fromSensor[i] ? MSG(s,d)22                  -> if23                      :: i == d -> toActuator[d] ! MSG(s,d)24                      :: else -> Edge2Fog[i] ! MSG(s,d)25                      fi }26      :: atomic { Fog2Edge[i] ? MSG(s,d) -> toActuator[d] ! MSG(s,d)27      fi28    od29 }30 proctype Fog (byte i) {31    byte j, x, s, d, p, q;32    do33    :: for (x : 0 .. (K-1)) {34       atomic { Edge2Fog[i*K+x] ? MSG(s,d)35                      -> j = d/K;36                         if37                         :: i == j -> Fog2Edge[d] ! MSG(s,d)38                         :: else -> if39                                     :: i > j -> p=K+j40                                     :: else -> p=K+j-141                                     fi42                                     Fog2Fog[(K-1)*i+p-K] ! MSG(s,d)43                         fi }44      }45    :: for (q : K .. (2*K-2)) {46         atomic { Fog2Fog[(mod(i-1-q+K,K)) + (q-K)*K] ? MSG(s,d)47                  -> Fog2Edge[d] ! MSG(s,d) }48      }49    od50 }51 init {52    byte i;53    for (i : 0 .. (K*(K-1))) {54         run Devices (i)55         run Edge(i) }56    for (i : 0 .. (K-1)) {57         run Fog (i) }58 }

## 6. Modeling with Acp

In view of the outcome attained with Spin/Promela simulation regarding the basic model, it is going to be proposed an alternative modeling by means of ACP. It is to be noted that ACP describes the functionality of the diverse sorts of entities taking part of a model by means of algebraic equations describing the behaviour of each of those, according to its set of rules, which do not include the notion of time.

In fact, this absence of time considerations changes the focus from quantitative analysis, as such being undertaken by means of the Spin/Promela simulation, to qualitative examinations, which permit to focus on diverse characteristics regarding the overall behaviour of the model without any time constraint. In this context, performance is measured in time units in the former, whereas alternative units are used in the latter [56].

ACP is an abstract process algebra whose main characteristic is to abstract away from the real nature of the entities being modeled, which allows to establish a generic high level approach to just focus on the behaviour of each entity being part of such a model [57]. This way, a generic IoT system may be modeled by first describing the behaviour of each type of entity, which in turn allows to undertake a model specification by setting all entities in a concurrent manner, which eventually may lead to carry out a model verification by inspecting the exterior behaviour of such a model, and then comparing to that of the real system [58].

In this context, verification may be achieved in ACP if algebraic equations describing the behaviour of both the model built up and the real system taken as a reference have the same string of actions and the same branching structure. If that is the case, they both are accounted to be rooted branching bisimilar, which is considered to be a sufficient condition to get a model verified [59].

It is to be noted that the way to face communications in ACP differ from that used in Promela, as the latter specifically declares the unidirectional channels where connections take place, whilst the former does not define any channels whatsoever, as those are the result of one end being able to communicate with the other end. This way, it is not necessary to set a mapping array to single out each unidirectional interfog channels, as all of them may be defined in a generic way thanks to the abstraction features of ACP.

Regarding the basic notions of ACP to implement models, it is to be said that there are two atomic actions, such as a given item Ω sending a generic message *d* through a particular channel *x*, which is described as $s_{\Omega ,x}\left(d\right)$ and a given item Ω reading a generic message *d* through a particular channel *x*, that being exposed by $r_{\Omega^{\prime },x^{\prime }}\left(d\right)$. In order to establish relationships among atomic actions, a bunch of operators are available, including a sequential one defined by ×, an alternate one given by +, a concurrent one denoted by ||, or a conditional one presented by the statement (True◃condition▹False) [60].

Furthermore, another operator called encapsulation, depicted by $\partial_{H}\left(\right)$, is necessary to carry out model specification, as it allows internal communications through a particular channel, given by $c_{\Omega ,x⟼x^{\prime },\Omega^{\prime }}\left(d\right)$ out of a sending action by an item Ω at the initial end of that channel, stated as *x*, such as $s_{\Omega ,x}\left(d\right)$ and a reading action by an item $\Omega^{\prime }$ at the final end of that channel, spotted as $x^{\prime }$, such as $r_{\Omega^{\prime },x^{\prime }}\left(d\right)$, which get both cancelled at that stage, so just the communication action prevails. Additionally, an extra operator named abstraction, exhibited by $\tau_{I}\left(\right)$, is needed to undertake model verification, as it permits internal actions and internal communications to be masked, whilst not affecting the external actions, hence unveiling the external behaviour of the model [61].

In order to better understand the ACP equations being cited in this section, it is to be said that each recursive equation describes a class of items, those being either end devices (H), edge nodes (E), fog nodes (F) or cloud nodes (G), where each of them may include a certain number of instances, these being defined by a summatory. The role of the items in the lower layer of each scenario is just being the source or destination of a traffic flow, that is, just sending or receiving data. However, the items in the upper layers of each scenario may execute different sets of actions if a traffic flow is received either through the lower ports or the upper ports, where the ports involved are selected through a summatory.

The sets of actions being run in each case are meant to guide the received traffic towards its intended destination by means of selecting the outgoing port having the optimal path to such a destination. Moreover, the diverse sets of actions are separated by the alternative operator (+) and each action within a given set contains sequential operations (×) indicating the order in which the relevant actions are run and some conditional statements (True◃condition▹False) with the target of finding the optimal path to destination, such as a fog element being the destination fog or an edge element being the destination edge, where the string of actions being executed after the condition depends on the completion of such a condition (those on the left hand side of the condition) or otherwise (those on the right hand side). Besides, the concurrent operator (∣∣) runs all entities involved in a simultaneous manner, thus making possible to obtain the sequence of events occurring in a particular scenario with the help of the rest of operators.

On the other hand, in order to portray the processing being carried out at the diverse remote computing facilities exposed in previous sections, each of them with its AI-based capacities, those are going to be exposed by using the Greek alphabet, such as $\alpha_{\mathrm{AI}}$ for AI-powered computations undertaken on an edge node, $\beta_{\mathrm{AI}}$ for AI-based calculations done on an fog node and $\gamma_{\mathrm{AI}}$ for AI-powered processing done on a cloud node. Moreover, it is to be said that the models presented do not incorporate any training schemes for those AI-based capacities, as it is supposed they are already trained for simplicity purposes. This way, the focus in the models may be set into the arithmetic operations to achieve an efficient path between any given source and any given destination.

In this context, some algebraic models are going to be proposed in an incremental manner. To start with, a core model is exposed, which only contains fog nodes, those represented by variable *F* and edge nodes, those expressed by variable *E*, according to the prerequisites established in previous section, such as *k* fogs and *k* edges per node, thus making up to $k^{2}$ edges overall. After that, a basic model is proposed, which adds up one single end device or host, given by variable *H*, per each of the edges, thus accounting for *k* end devices per edge node, which in turn makes up to $k^{2}$ end devices overall. It is to be remarked that this is the model designed with Promela and executed with Spin in the last section.

Besides, an extended model is exhibited, which incorporates *k* end devices per edge, thus making up to $k^{2}$ end devices per fog and accounting for $k^{3}$ overall. Moreover, an enhanced model is shown, which incorporates just 2 cloud nodes for redundancy purposes, given by variable *G*, such that each fog has a direct link to each of those. Additionally, a full model could also be built up by including sensors and actuators, even though it is not going to be depicted as it might be easily deducted following the same pattern as the extended one, in a way that it might account for *k* sensors and *k* actuators connected per end device, which might do for $k^{2}$ for each type per edge, $k^{3}$ for each kind per fog and $k^{4}$ for each sort overall.

As in the previous section, it is to be reminded that all those models are based on key arithmetic tools, such as integer division and modular arithmetic. However, integer division in excess is also being used so as to find out the ports related to unidirectional interfog communications, as quoted in Equations (3) and (4). Therefore, it is interesting to cite some alternative ways to calculate them, so Equation (7) presents integer division, Equation (8) exposes modulus *k* and Equation (9) exhibits integer division in excess.

(7) $$\mathrm{int}\left(a\;/\;b\right)\;=\;\left\lfloor a\;/\;b\right\rfloor \;=\;\left(a-a_{|b}\right)\;/\;b$$

(8) $$a\;\mathrm{mod}\;b\;=\;a_{|b}\;=\;{\left(a_{|b}+b\right)}_{|b}$$

(9) $$\left\lceil a\;/\;b\right\rceil \;=\;-\left\lfloor -a\;/\;b\right\rfloor \;=\;-\left(a_{|b}-a\right)\;/\;b$$

It is to be noted that in all models presented, the items in the lowest layer just sends raw data (d) or receives processed data (e), whilst the items in the upper layers may carry out two chain of actions. The first one is related to the reception of a raw message through any of its downlink ports, followed by dealing with it by either processing it and sending it back to an item in the lower layer on the way to its final destination, or otherwise, forwarding it to an item in the upper layer for it to take charge of it. The second one is related to the reception of a processed message through any of its uplink ports, followed by processing it and sending it back to an item in the lower layer on the way to its final destination.

Regarding optimization in communications, the arithmetic framework proposed makes use of integer divisions by *k* for an item located in any layer to find the element where it is hanging on to, as well as modular arithmetic in order to find out the port looking at either the source host or the destination host, depending whether the segment considered within the path is upwards or downwards.

Therefore, the optimization of resources when using the presented models are based in the use of integer divisions and modular arithmetic to achieve traffic forwarding in an optimal way by just applying arithmetic operations, as opposed to getting it by means of looking up into the forwarding tables of the relevant devices. The main benefit of the former compared to the latter are the simplicity of operations, that leading to achieve shorter response times when finding out the proper forwarding route, even though the latter may be used in any condition, whereas the former requires the deployment of the framework proposed to get optimal results, or otherwise suboptimal outcomes arise.

### 6.1. Core Scenario: Edge-Fog

**Figure 3:**
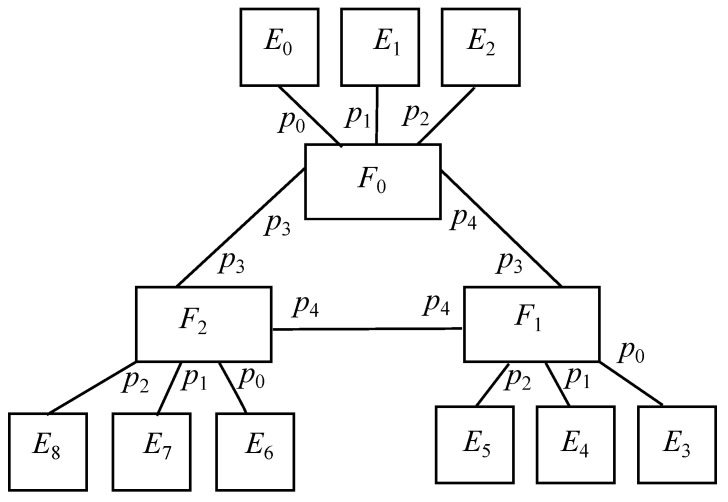
Core scenario: Edge-Fog for k=3.

In this case, there are only fog nodes (F) and edge nodes (E), as exposed in Figure 3, where the former has *k* downlink ports, those ranging {0⋯k−1}, and k−1 uplink ports, those going {k⋯2k−2}, whilst the latter shows just 1 uplink port, labeled as 0. As stated before, there are *k* fog nodes and *k* edge nodes per fog, resulting in $k^{2}$ overall.

Furthermore, as explained in previous sections, all edge nodes connected to a fog node share the same result of their integer division by *k*, which just happens to be the fog node identifier. On the other hand, the downlink ports of all fog nodes are congruent modulo *k* with the edge node connected to each port, whereas the uplink ports of all fog nodes may be calculated by Equations (3) and (4), which makes use of integer division in excess to achieve both ends of each unidirectional interfog channel.

With all that in mind, Equation (10) describes the behaviour of a generic edge node $E_{\epsilon }$ and Equation (11) does it for a generic fog node $F_{\phi }$. In this sense, it is to be noted that atomic actions bear two parameters, such as the former is the device identifier and the latter is the port involved, whilst ϵ and ϕ are the identifier of each one of the edge nodes and fog nodes, respectively.

In addition, the only AI-based processing is carried out at fog premises, branded as $\beta_{\mathrm{AI}}$, because edge devices are just the source (*a*) or destination (*b*) of all messages going through the system. Furthermore, generic raw data are labeled as *d* prior to being computed by an AI-based processing, whereas they are done as *e* after that point. Besides, the sending end of an interfog link is found out by Equation (3), where the source fog is branded as $i=\left\lfloor a\;/\;k\right\rfloor$, whereas destination fog is done as $j=\left\lfloor b\;/\;k\right\rfloor$, even though the destination end is irrelevant in this algebraic model, as the destination fog just listens to all uplink ports and sends any received message towards its proper destination edge.

(10) $$E_{\epsilon }=\sum_{\epsilon =0}^{k^{2}-1}\left(s_{E_{\epsilon },0}\left(d\right)+r_{E_{\epsilon },0}\left(e\right)\right)\times E_{\epsilon }$$

(11) $$\begin{array}{c} F_{\phi }=\sum_{\phi =0}^{k-1}(\sum_{x=0}^{k-1}r_{F_{\phi },x}\left(d\right)\times \beta_{\mathrm{AI}}\times \left(s_{F_{\phi },b_{|k}}\left(e\right)◃\;\phi =\left\lfloor b\;/\;k\right\rfloor \;▹s_{F_{\phi },k+j-\left\lceil \left\lfloor \frac{j}{i+1}\right\rfloor \;/\;k\right\rceil }\left(e\right)\right)+\\ \sum_{x=k}^{2k-2}r_{F_{\phi },x}\left(e\right)\times s_{F_{\phi },b_{|k}}\left(e\right))\times F_{\phi } \end{array}$$

At this stage, all entities may be executed in a concurrent fashion, where the encapsulation operator restricts the effects of non-determinism. Actually, such an operator enables all internal communications, which reveals the sequence of events occurring in this model, as seen in Equation (12). It is to be reminded that in this case, the edge nodes are the source and sink of all messages, so they do not take part of the internal model, which is now restricted just to fog nodes. Moreover, ∅ indicates that no action is taken within a conditional statement. Besides, the statement $c_{source\_item,source\_port⟼destination\_port,destination\_item}$ describes the elements of each internal communication occurred within the model.

(12) $$\begin{array}{c} \sum_{\phi =0}^{k-1}\partial_{H}\left(F_{\phi }\right)=(\sum_{x=0}^{k-1}r_{F_{\phi },x}\left(d\right)\times \beta_{\mathrm{AI}}\times \\ \left(\emptyset ◃\;\phi =\left\lfloor b\;/\;k\right\rfloor \;▹c_{F_{i},k+j-\left\lceil \left\lfloor \frac{j}{i+1}\right\rfloor \;/\;k\right\rceil ⟼k+i-\left\lceil \left\lfloor \frac{i}{j+1}\right\rfloor \;/\;k\right\rceil ,F_{j}}\left(e\right)\right)\times s_{F_{\phi },b_{|k}}\left(e\right))\times \partial_{H}\left(F_{\phi }\right) \end{array}$$

At this point, the model have been specified and the application of the abstraction operator on the aforesaid specification may reveal its external behaviour, as that operator will mask both internal communications and internal actions, hence prevailing only the external actions.

(13) $$\sum_{\phi =0}^{k-1}\tau_{I}\left(\partial_{H}\left(F_{\phi }\right)\right)=\sum_{x=0}^{k-1}r_{F_{\phi },x}\left(d\right)\times s_{F_{\phi },b_{|k}}\left(e\right)\times \tau_{I}\left(\partial_{H}\left(F_{\phi }\right)\right)$$

On the other hand, the external behaviour of the real system consists of receiving some raw data (d) from a source edge from an incoming link, and after processing them and turning them into processed data (e), it is send over to a destination edge towards an outgoing link, as shown in Equation (14).

(14) $$X=r_{IN}\left(d\right)\times s_{OUT}\left(e\right)\times X$$

Confronting Equation (13) with Equation (14), it may seem obvious that they are both recursive equations being multiplied by the same factors, even though the nomenclature of the incoming and outgoing channels may differ. Hence, it may be stated that both exquations are rooted branching bisimilar, as they exhibit the same actions, along with the same branching structure, which induces the application of Equation (15).

(15) $$\sum_{\phi =0}^{k-1}\tau_{I}\left(\partial_{H}\left(F_{\phi }\right)\right)⟷X$$

Therefore, that is a sufficient condition to have a model verified; hence, the core model proposed in ACP may be considered as verified.

### 6.2. Basic Scenario: Oneenddevice-Edge-Fog

**Figure 4:**
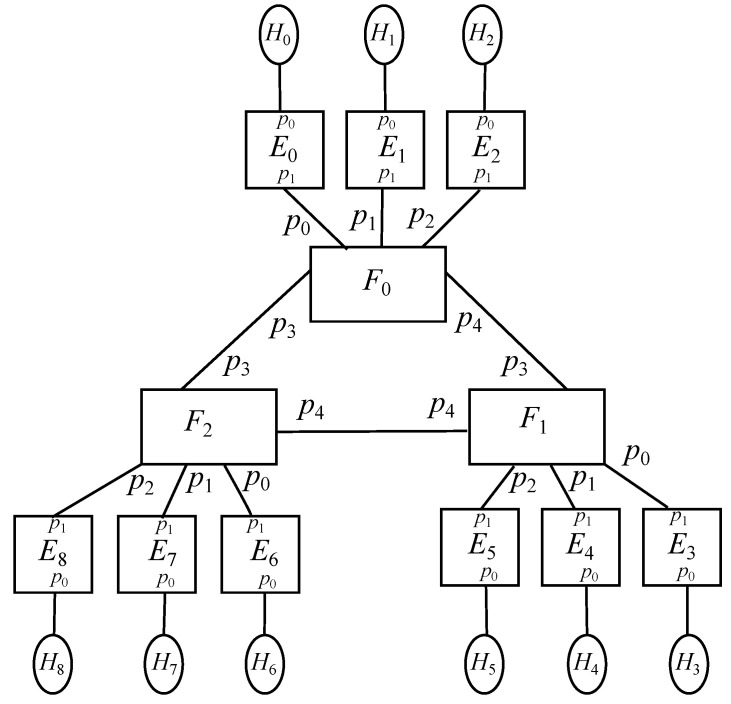
Basic scenario: OneEndDevice-Edge-Fog for k=3.

Taking the previous model as a core, an extra end device is connected to every edge node, which are represented by *H*. This way, edge nodes will have a downlink port to connect to its associate end device and an uplink port to connect to its associated fog node, where the former is identified by 0 and the latter does it by 1. On the contrary, the only port located in all end devices is identified by 0. It is to be reminded that this is the basic model studied above in the Spin/Promela code and it is depicted in Figure 4.

In this case, there are *k* fogs, $k^{2}$ edges and $k^{2}$ end devices, where the latter just generate messages (a) and receive messages (b), whereas the other two construct the modeled system. Taking that into account, Equation (16) denotes the behaviour of a generic end device $H_{\eta }$, Equation (17) describes it for a generic edge node $E_{\epsilon }$ and Equation (18) states it for a generic fog node $F_{\phi }$, which matches Equation (11). Furthermore, AI-powered tasks are undertaken on edge nodes, labeled as $\alpha_{\mathrm{AI}}$, as well as on fog node, named as $\beta_{\mathrm{AI}}$.

(16) $$H_{\eta }=\sum_{\eta =0}^{k^{2}-1}\left(s_{H_{\eta },0}\left(d\right)+r_{H_{\eta },0}\left(e\right)\right)\times H_{\eta }$$

(17) $$\begin{array}{c} E_{\epsilon }=\sum_{\epsilon =0}^{k^{2}-1}\left(r_{E_{\epsilon },0}\left(d\right)\times \left(\alpha_{\mathrm{AI}}\times s_{E_{\epsilon },0}\left(e\right)◃\;\epsilon =b\;▹s_{E_{\epsilon },1}\left(d\right)\right)+r_{E_{\epsilon },1}\left(e\right)\times s_{E_{\epsilon },0}\left(e\right)\right)\times E_{\epsilon } \end{array}$$

(18) $$\begin{array}{c} F_{\phi }=\sum_{\phi =0}^{k-1}(\sum_{x=0}^{k-1}r_{F_{\phi },x}\left(d\right)\times \beta_{\mathrm{AI}}\times \left(s_{F_{\phi },b_{|k}}\left(e\right)◃\;\phi =\left\lfloor b\;/\;k\right\rfloor \;▹s_{F_{\phi },k+j-\left\lceil MM\left\lfloor \frac{j}{i+1}\right\rfloor \;/\;k\right\rceil }\left(e\right)\right)+\\ \sum_{x=k}^{2k-2}r_{F_{\phi },x}\left(e\right)\times s_{F_{\phi },b_{|k}}\left(e\right))\times F_{\phi } \end{array}$$

At the stage, the encapsulation operator reveals the sequence of events taking place in the model, as seen in Equation (19). It is to be remarked that in this case, the end devices are the source and sink of all messages, labeled as *a* and *b*, respectively, so they do not participate in the internal model, which is now restricted just to edge nodes and fog nodes. Furthermore, source edge is identified by $E_{a}$, whilst destination edge is done by $E_{b}$, as there are just one end device per edge. On the other hand, source fog is labeled by $F_{\left\lfloor a\;/\;k\right\rfloor }$, whereas destination fog is done by $F_{\left\lfloor b\;/\;k\right\rfloor }$.

(19) $$\begin{array}{c} \sum_{\epsilon =0}^{k^{2}-1}\sum_{\phi =0}^{k-1}\partial_{H}\left(E_{\epsilon }\left|\right|F_{\phi }\right)=(r_{E_{a},0}\left(d\right)\times (\alpha_{\mathrm{AI}}◃\;a=b\;▹(c_{E_{a},1⟼a_{|k},F_{\left\lfloor a\;/\;k\right\rfloor }}\left(d\right)\times \beta_{\mathrm{AI}}\times \\ \left(\emptyset ◃\;\left\lfloor a\;/\;k\right\rfloor =\left\lfloor b\;/\;k\right\rfloor \;▹c_{F_{i},k+j-\left\lceil \left\lfloor \frac{j}{i+1}\right\rfloor \;/\;k\right\rceil ⟼k+i-\left\lceil \left\lfloor \frac{i}{j+1}\right\rfloor \;/\;k\right\rceil ,F_{j}}\left(e\right)\right)\times \\ c_{F_{\left\lfloor b\;/\;k\right\rfloor },b_{|k}⟼1,E_{\left\lfloor b\;/\;k\right\rfloor }}\left(e\right))\times s_{E_{b},0}\left(e\right)))\times \partial_{H}\left(E_{\epsilon }\left|\right|F_{\phi }\right) \end{array}$$

At this point, the abstraction operator unveils the external behaviour of the model, as shown in Equation (20).

(20) $$\sum_{\epsilon =0}^{k^{2}-1}\sum_{\phi =0}^{k-1}\tau_{I}\left(\partial_{H}\left(E_{\epsilon }\left|\right|F_{\phi }\right)\right)=r_{E_{a},0}\left(d\right)\times s_{E_{b},0}\left(e\right)\times \tau_{I}\left(\partial_{H}\left(E_{\epsilon }\left|\right|F_{\phi }\right)\right)$$

On the other hand, the external behaviour of the real system matches that cited in Equation (14).

Comparing Equation (20) with Equation (14), the same reasoning applies herein, which induces the application of Equation (21).

(21) $$\sum_{\epsilon =0}^{k^{2}-1}\sum_{\phi =0}^{k-1}\tau_{I}\left(\partial_{H}\left(E_{\epsilon }\left|\right|F_{\phi }\right)\right)⟷X$$

Hence, this is a sufficient condition to get a model verified; thus, the basic model proposed in ACP may be considered as verified.

### 6.3. Extended Scenario: Multipleenddevice-Edge-Fog

**Figure 5:**
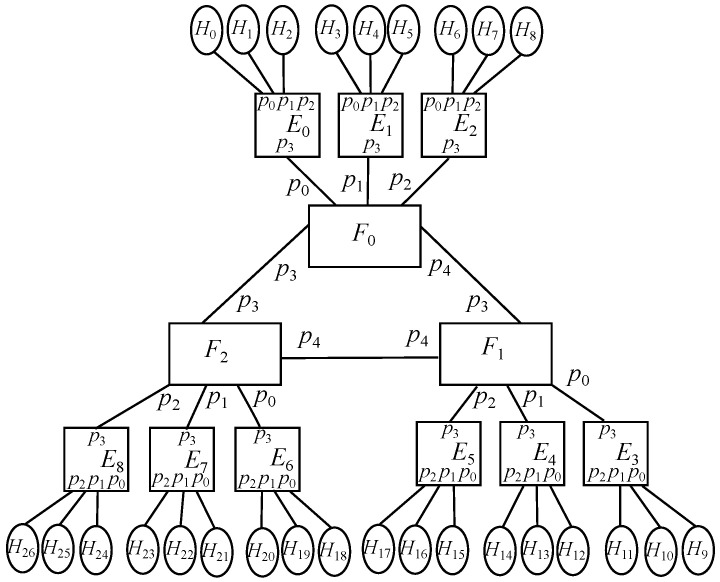
Extended scenario: MultipleEndDevices-Edge-Fog for k=3.

The previous model may be easily extended by adding up *k* end devices per edge node, resulting in $k^{2}$ per fog node and $k^{3}$ overall. Hence, end devices may calculate the edge identifier they are hanging on by applying an integer division by *k*, whilst the fog node they have above is found out with an integer division by $k^{2}$. On the other hand, arithmetic modulo *k* may find the port of an edge device connected to an end device, whereas arithmetic modulo $k^{2}$ does it for the path from a fog node aimed at a given end device. Regardless, Figure 5 exposes the overall picture.

The equations describing the types of entities involved are Equation (22) for end devices (H), which accounts for more items than Equation (16), Equation (23) for edge nodes (F), whose difference with Equation (17) is that now an edge has *k* downlink ports, going from 0 to k−1, and 1 uplink port identified by *k*, and also Equation (24) for fog devices (F), which is analogous to Equation (18).

(22) $$H_{\eta }=\sum_{\eta =0}^{k^{3}-1}\left(s_{H_{\eta },0}\left(d\right)+r_{H_{\eta },0}\left(e\right)\right)\times H_{\eta }$$

(23) $$\begin{array}{c} E_{\epsilon }=\sum_{\epsilon =0}^{k^{2}-1}\left(\sum_{y=0}^{k-1}r_{E_{\epsilon },y}\left(d\right)\times \left(\alpha_{\mathrm{AI}}\times s_{E_{\epsilon },b_{|k}\left(e\right)}◃\;\epsilon =\left\lfloor b\;/\;k\right\rfloor \;▹s_{E_{\epsilon },k}\left(d\right)\right)+r_{E_{\epsilon },k}\left(e\right)\times s_{E_{\epsilon },b_{|k}}\left(e\right)\right)\times E_{\epsilon } \end{array}$$

(24) $$\begin{array}{c} F_{\phi }=\sum_{\phi =0}^{k-1}(\sum_{x=0}^{k-1}r_{F_{\phi },x}\left(d\right)\times \beta_{\mathrm{AI}}\times \left(s_{F_{\phi },b_{|k}}\left(e\right)◃\;\phi =\left\lfloor b\;/\;k^{2}\right\rfloor \;▹s_{F_{\phi },k+j-\left\lceil \left\lfloor \frac{j}{i+1}\right\rfloor \;/\;k\right\rceil }\left(e\right)\right)+\\ \sum_{x=k}^{2k-2}r_{F_{\phi },x}\left(e\right)\times s_{F_{\phi },b_{|k}}\left(e\right))\times F_{\phi } \end{array}$$

At this moment, the encapsulation operator is applied so as to obtain the sequence of events occurring in the model, as exposed in Equation (25).

(25) $$\begin{array}{c} \sum_{\epsilon =0}^{k^{2}-1}\sum_{\phi =0}^{k-1}\partial_{H}\left(E_{\epsilon }\left|\right|F_{\phi }\right)=(r_{E_{a},a_{|k}}\left(d\right)\times (\alpha_{\mathrm{AI}}◃\;\left\lfloor a\;/\;k\right\rfloor =\left\lfloor b\;/\;k\right\rfloor \;▹(c_{E_{a},k⟼a_{|k},F_{\left\lfloor a\;/\;k^{2}\right\rfloor }}\left(d\right)\times \beta_{\mathrm{AI}}\times \\ \left(\emptyset ◃\;\left\lfloor a\;/\;k^{2}\right\rfloor =\left\lfloor b\;/\;k^{2}\right\rfloor \;▹c_{F_{i},k+j-\left\lceil \left\lfloor \frac{j}{i+1}\right\rfloor \;/\;k\right\rceil ⟼k+i-\left\lceil \left\lfloor \frac{i}{j+1}\right\rfloor \;/\;k\right\rceil ,F_{j}}\left(e\right)\right)\times \\ c_{F_{\left\lfloor b\;/\;k^{2}\right\rfloor },b_{|k}⟼k,E_{\left\lfloor b\;/\;k\right\rfloor }}\left(e\right))\times s_{E_{b},b_{|k}}\left(e\right)))\times \partial_{H}\left(E_{\epsilon }\left|\right|F_{\phi }\right) \end{array}$$

At that point, the abstraction operator reveals the external behaviour of the model, as exhibited in Equation (26).

(26) $$\sum_{\epsilon =0}^{k^{2}-1}\sum_{\phi =0}^{k-1}\tau_{I}\left(\partial_{H}\left(E_{\epsilon }\left|\right|F_{\phi }\right)\right)=r_{E_{a},a_{|k}}\left(d\right)\times s_{E_{b},b_{|k}}\left(e\right)\times \tau_{I}\left(\partial_{H}\left(E_{\epsilon }\left|\right|F_{\phi }\right)\right)$$

Moreover, the external behaviour of the real system matches that quoted in Equation (14), which results to be rooted branching bisimilar to Equation (26), thus that is a sufficient condition to get a model verified; thus, the basic model proposed in ACP may be considered as verified.

### 6.4. Enhanced Scenario: Multipleenddevices-Edge-Fog-Cloud Scenario

**Figure 6:**
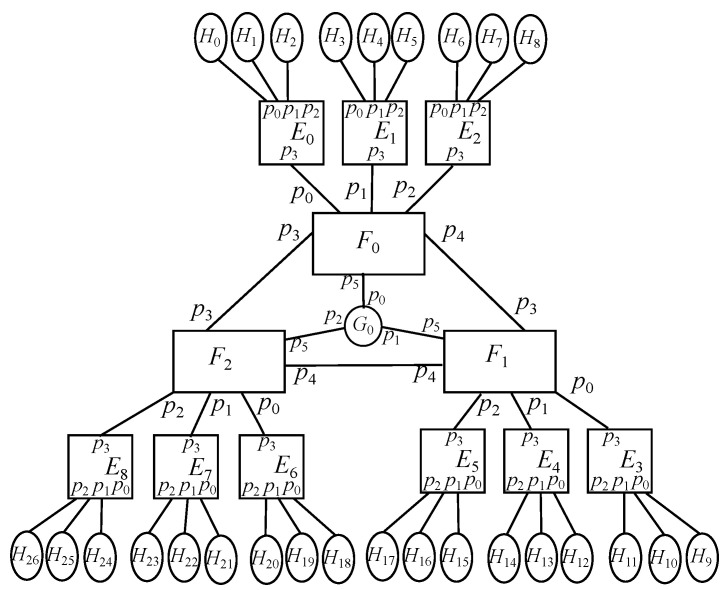
Enhanced scenario: MultipleEndDevices-Edge-Fog-Cloud for k=3.

The previous model may be enhanced by implementing a redundant cloud system, which are represented by *G*, in a way that each fog node will use link 2k−1 to reach the cloud node for backup and storage purposes. On the other hand, the links in the cloud facilities match those of the fog nodes receiving such interconnections. Besides, it is to be noted that the cloud nodes will have AI-powered processing, that being denoted as $\gamma_{AI}$. Putting all together, Figure 6 depicts the overall picture.

In this context, Equation (22) still applies for end devices, whilst Equation (23) still do for edge nodes. With respect to fog nodes, Equation (27) takes Equation (24) as a foundation, and from there on, it includes communication with the cloud whenever it gets some traffic flow in order to implement backup policies regarding processing or storage. Additionally, Equation (28) presents the model for cloud node.

(27) $$\begin{array}{c} F_{\phi }=\sum_{\phi =0}^{k-1}((\sum_{x=0}^{k-1}r_{F_{\phi },x}\left(d\right)\times \beta_{\mathrm{AI}}\times \left(s_{F_{\phi },2k-1}\left(e\right)+r_{F_{\phi },2k-1}\left(e\right)\right)\times \\ \left(s_{F_{\phi },b_{|k}}\left(e\right)◃\;\phi =\left\lfloor b\;/\;k^{2}\right\rfloor \;▹s_{F_{\phi },k+j-\left\lceil \left\lfloor \frac{j}{i+1}\right\rfloor \;/\;k\right\rceil }\left(e\right)\right)+\sum_{x=k}^{2k-2}r_{F_{\phi },x}\left(e\right)\times s_{F_{\phi },b_{|k}}\left(e\right))+)\times F_{\phi } \end{array}$$

(28) $$G_{\zeta }=\left(\sum_{z=0}^{k-1}r_{G_{\zeta },z}\left(d\right)\times \gamma_{AI}\times r_{G_{\zeta },z}\left(e\right)\right)\times G_{\zeta }$$

At this stage, the encapsulation operator is applied so as to achieve the sequence of events happening in the model, as shown in Equation (29).

(29) $$\begin{array}{c} \sum_{\epsilon =0}^{k^{2}-1}\sum_{\phi =0}^{k-1}\partial_{H}\left(E_{\epsilon }\left|\right|F_{\phi }\left|\right|G_{\zeta }\right)=(r_{E_{a},a_{|k}}\left(d\right)\times (\alpha_{\mathrm{AI}}◃\;\left\lfloor a\;/\;k\right\rfloor =\left\lfloor b\;/\;k\right\rfloor \;▹(c_{E_{a},k⟼a_{|k},F_{\left\lfloor a\;/\;k^{2}\right\rfloor }}\left(d\right)\times \beta_{\mathrm{AI}}\times \\ \left(c_{F_{\left\lfloor b\;/\;k^{2}\right\rfloor },2k-1⟼\left\lfloor b\;/\;k^{2}\right\rfloor ,G_{\zeta }}\left(e\right)\times \gamma_{AI}\times c_{G_{\zeta },\left\lfloor b\;/\;k^{2}\right\rfloor ⟼2k-1,F_{\left\lfloor b\;/\;k^{2}\right\rfloor }}\right)\times \\ \left(\emptyset ◃\;\left\lfloor a\;/\;k^{2}\right\rfloor =\left\lfloor b\;/\;k^{2}\right\rfloor \;▹c_{F_{i},k+j-\left\lceil \left\lfloor \frac{j}{i+1}\right\rfloor \;/\;k\right\rceil ⟼k+i-\left\lceil \left\lfloor \frac{i}{j+1}\right\rfloor \;/\;k\right\rceil ,F_{j}}\left(e\right)\right)\times \\ c_{F_{\left\lfloor b\;/\;k^{2}\right\rfloor },b_{|k}⟼k,E_{\left\lfloor b\;/\;k\right\rfloor }}\left(e\right)\times s_{E_{b},b_{|k}}\left(e\right)))\times \partial_{H}\left(E_{\epsilon }\left|\right|F_{\phi }\left|\right|G_{\zeta }\right) \end{array}$$

At that point, the abstraction operator unveils the external behaviour of the model, as exhibited in Equation (30).

(30) $$\sum_{\epsilon =0}^{k^{2}-1}\sum_{\phi =0}^{k-1}\tau_{I}\left(\partial_{H}\left(E_{\epsilon }\left|\right|F_{\phi }\left|\right|G_{\zeta }\right)\right)=r_{E_{a},a_{|k}}\left(d\right)\times s_{E_{b},b_{|k}}\left(e\right)\times \tau_{I}\left(\partial_{H}\left(E_{\epsilon }\left|\right|F_{\phi }\left|\right|G_{\zeta }\right)\right)$$

Besides, the external behaviour of the real system matches that cited in Equation (14), which occurs to be rooted branching bisimilar to Equation (30); hence, this is a sufficient condition to have a model verified; thus, the basic model proposed in ACP may be considered as verified.

### 6.5. How the Model Works

It is to be considered that the arithmetic model proposed is aimed at speeding up the times for routing and switching by means of applying simple arithmetic operations, such as integer divisions and modular arithmetic, as opposed to using the traditional routing and switching strategies where the next hop of a destination is searched by means of looking up into a certain table with a number of entries.

The optimal strategy regarding performance in this context may depend on which one is faster, either the arithmetic operations or the searching operations, according to the hardware and software resources available and how those operations are implemented. In this sense, the key point when assessing whether the arithmetic strategy, based on integer divisions and modular arithmetic, performs better than the traditional searching algorithms when dealing with routing and switching will basically depend on how those different approaches are coded into the ASIC hardware and the software complements being used, although the arithmetic approach seems to be more efficient in theory.

In other words, making the necessary arithmetic calculations to go from a source host to a destination host may seem faster beforehand than searching through lookup tables with many registers on them; hence, it may appear that the arithmetic framework might be more efficient than searching into the forwarding tables. Nonetheless, the way those operations are implemented in hardware and software will definitely influence which method is the most efficient. However, it is to be noted that the arithmetic operations are only useful for the specific framework proposed, with the appropriate number of elements on each layer, whilst the searching operations do apply to any type of topology.

Focusing on the basics of the model described, and keeping in mind that *a* represents the source end device and *b* does the destination one, for any value of parameter *k*, it is to be reminded that intraedge communication occurs when the source edge ($E_{\left\lfloor a\;/\;k\right\rfloor }$) and the destination edge $\left(E_{\left\lfloor b\;/\;k\right\rfloor }\right)$ match, such that $E_{\left\lfloor a\;/\;k\right\rfloor }=E_{\left\lfloor b\;/\;k\right\rfloor }$, whilst intrafog communications happens when the intraedge condition is not met, accounting for $E_{\left\lfloor a\;/\;k\right\rfloor }\neq E_{\left\lfloor b\;/\;k\right\rfloor }$, although at the same time the source fog ($F_{\left\lfloor a\;/\;k^{2}\right\rfloor }$) and the destination fog ($F_{\left\lfloor b\;/\;k^{2}\right\rfloor }$) match, such that $F_{\left\lfloor a\;/\;k^{2}\right\rfloor }=F_{\left\lfloor b\;/\;k^{2}\right\rfloor }$, whereas interfog communications take place when the intrafog condition is not met, such that $F_{\left\lfloor a\;/\;k^{2}\right\rfloor }\neq F_{\left\lfloor b\;/\;k^{2}\right\rfloor }$, which obviously involves $E_{\left\lfloor a\;/\;k\right\rfloor }\neq E_{\left\lfloor b\;/\;k\right\rfloor }$.

Regarding the ports involved in each case scenario, for intraedge communications the edge source port is given by $a_{|k}$ and the edge destination port is done by $b_{|k}$. Besides, for intrafog communications the fog source port is given ${\left\lfloor a\;/\;k\right\rfloor }_{|k}$ and the fog destination port is done by ${\left\lfloor b\;/\;k\right\rfloor }_{|k}$, whilst the edge downlink source and destination ports are the same as in the intraedge case and the edge uplink source and destination ports are *k* in both cases. Moreover, for interfog communications the fog uplink source port was cited in Equation (3) and the fog uplink destination port was quoted in Equation (4), whilst the fog downlink source and destination ports match those given in the intrafog case, and the same happens with the ports related to the edge nodes.

In that sense, some examples may be quoted so as to test how the arithmetic framework works. For this matter, let us take the extended model (studied in Section 6.3) with parameter k=3 and let us show a test of intraedge, intrafog and interfog scenarios. That way, the optimal path between any given pair of end devices are completely defined, quoting all intermediate nodes being part of that path and the downlink and uplink ports being used on them.

- Intraedge: source host a=4, destination host b=5. First of all, check the intraedge condition: $E_{\left\lfloor a\;/\;k\right\rfloor }=E_{\left\lfloor b\;/\;k\right\rfloor }\to E_{\left\lfloor 4\;/\;3\right\rfloor }=E_{\left\lfloor 5\;/\;3\right\rfloor }=E_{1}$ (common edge node $E_{1}$), where its edge source downlink port is $a_{|k}=4_{|3}=1$ and its edge destination donwlink port is $b_{|k}=5_{|3}=2$.
- Intrafog: source host a=4, destination host b=8. To start with, check the intraedge condition: $E_{\left\lfloor a\;/\;k\right\rfloor }=E_{\left\lfloor b\;/\;k\right\rfloor }\to E_{\left\lfloor 4\;/\;3\right\rfloor }=E_{1}\neq E_{\left\lfloor 8\;/\;3\right\rfloor }=E_{2}$ (source edge node $E_{1}$, whilst destination edge node $E_{2}$). Then, check the intrafog condition: $F_{\left\lfloor a\;/\;k^{2}\right\rfloor }=F_{\left\lfloor b\;/\;k^{2}\right\rfloor }\Rightarrow F_{\left\lfloor 4\;/\;3^{2}\right\rfloor }=F_{\left\lfloor 8\;/\;3^{2}\right\rfloor }=F_{0}$ (common fog node $F_{0}$), where its fog source downlink port is ${\left\lfloor a\;/\;k\right\rfloor }_{|k}={\left\lfloor 4\;/\;3\right\rfloor }_{|3}=1_{|3}=1$ and its fog destination downlink port is ${\left\lfloor b\;/\;k\right\rfloor }_{|k}={\left\lfloor 8\;/\;3\right\rfloor }_{|3}=2_{|3}=2$. Furthermore, source edge node is $E_{\left\lfloor a\;/\;k\right\rfloor }=E_{\left\lfloor 4\;/\;3\right\rfloor }=E_{1}$, where its edge source downlink port is $a_{|k}=4_{|3}=1$, whilst its edge source uplink port is k=3, and destination edge node is $E_{\left\lfloor b\;/\;k\right\rfloor }=E_{\left\lfloor 8\;/\;3\right\rfloor }=E_{2}$, where its edge destination downlink port is $b_{|k}=8_{|3}=2$, whilst its edge destination uplink port is k=3.
- Interfog: source host a=4, destination host b=9. To begin with, check the intraedge condition: $E_{\left\lfloor a\;/\;k\right\rfloor }=E_{\left\lfloor b\;/\;k\right\rfloor }\to E_{\left\lfloor a\;/\;k\right\rfloor }\neq E_{\left\lfloor b\;/\;k\right\rfloor }\to E_{\left\lfloor 4\;/\;3\right\rfloor }=E_{1}\neq E_{\left\lfloor 9\;/\;3\right\rfloor }=E_{3}$ (there is no common edge node). In turn, check the intrafog condition: $F_{\left\lfloor a\;/\;k^{2}\right\rfloor }=F_{\left\lfloor b\;/\;k^{2}\right\rfloor }\Rightarrow F_{\left\lfloor 4\;/\;3^{2}\right\rfloor }=F_{0}\neq F_{\left\lfloor 9\;/\;3^{2}\right\rfloor }=F_{1}$ (source fog node $F_{0}$, whilst destination fog node $F_{1}$), where the fog source uplink port in $F_{0}$ is $k+\left\lfloor b\;/\;k^{2}\right\rfloor +\left\lfloor -\left\lfloor \left\lfloor b\;/\;k^{2}\right\rfloor /\left(\left\lfloor a\;/\;k^{2}\right\rfloor +1\right)\right\rfloor /k\right\rfloor =3+1+\left\lfloor -\left\lfloor 1\;/\;\left(0+1\right)\right\rfloor /3\right\rfloor =4$ and the fog destination uplink port in $F_{1}$ is $k+\left\lfloor a\;/\;k^{2}\right\rfloor +\left\lfloor -\left\lfloor \left\lfloor a\;/\;k^{2}\right\rfloor /\left(\left\lfloor b\;/\;k^{2}\right\rfloor +1\right)\right\rfloor /k\right\rfloor =3+0+\left\lfloor -\left\lfloor 0\;/\;\left(1+1\right)\right\rfloor /3\right\rfloor =3$, whereas fog source downlink port is ${\left\lfloor a\;/\;k\right\rfloor }_{|k}={\left\lfloor 4\;/\;3\right\rfloor }_{|3}=1_{|3}=1$ and fog destination downlink port is ${\left\lfloor b\;/\;k\right\rfloor }_{|k}={\left\lfloor 9\;/\;3\right\rfloor }_{|3}=9_{|3}=0$. Furthermore, source edge is $E_{\left\lfloor a\;/\;k\right\rfloor }=E_{\left\lfloor 4\;/\;3\right\rfloor }=E_{1}$, where its edge source downlink port is $a_{|k}=4_{|3}=1$, whilst its edge source uplink port is k=3, and destination edge is $E_{\left\lfloor b\;/\;k\right\rfloor }=E_{\left\lfloor 9\;/\;3\right\rfloor }=E_{3}$, where its edge destination downlink port is $b_{|k}=9_{|3}=0$, whilst its edge destination uplink port is k=3.

## 7. Conclusions

In this paper, we undertook the modeling of a generic edge computing infrastructure for IoT devices. To start with, a small introduction about the application of computational intelligence to IoT environments has been proposed, as well as some notion about FDT have been stated.

All those points are being implemented when dealing with IoT ecosystems, which are rising exponentially in recent times and most analyst forecast even higher growth rates in the coming years, in fields as IIoT, VANET or IoT-based healthcare. However, focusing in the application of computational intelligence to IoT, it may be considered that such devices have limited computing resources; hence, they need to outsource their computing tasks to a remote computing node.

In this sense, edge computing seems to be the key player, as those are located the closest to IoT devices, whereas fog nodes may be used as an alternative computing scheme, or otherwise, being higher in the remote computing hierarchy, whilst cloud nodes may be employed as a backup system for them all.

The strategy being followed here in is to situate edge nodes in the lower level, thus being directly in touch with IoT devices, whereas fog nodes are just above the edge ones in the hierarchy, hence providing them with backup facilities for processing or storage. In addition, cloud nodes are above fog nodes in the hierarchy, thus delivering backup services to fog facilities.

In this context, a generic model has been proposed herein, in a way that a bunch of fog servers are interconnected by means of a full mesh network topology with a link to a cloud node for backup necessities. Additionally, each of those fog servers has a certain number of edge nodes hanging on them, which at the same time are providing services to IoT items, playing the part of end devices.

This generic model has been formally described by means of two different FDT, such as Spin/Promela and ACP, where the former might be seen as a timed FDT and the latter may be considered as a timeless one. In this sense, a Spin/Promela model was designed although an explosion of states occurred with just a restricted number of devices. This situation led to the adoption of an ACP approach, which allowed us to obtain generic algebraic models related to the scenarios proposed, where all those were duly verified.

## Figures and Tables

**Table 1:** Mapping of fogs and their uplink ports in case k=4.

Source Fog: *i*	Source Port: *p*	Destination Fog: *j*	Destination Port: *q*	Mapping Array: *m* (Index and Its Value)
0	4	1	4	0
0	5	2	4	1
0	6	3	4	2
1	4	0	4	3
1	5	2	5	4
1	6	3	5	5
2	4	0	5	6
2	5	1	5	7
2	6	3	6	8
3	4	0	6	9
3	5	1	6	10
3	6	2	6	11

**Table 2:** Outcome of the Spin simulation cited.

K	State-Vector (Bytes)	Depth Reached	States Stored	States Matched
2	276	120	1,179,021 $\times 10^{6}$	1,220,492 $\times 10^{6}$
3	588	236	78,664,413 $\times 10^{6}$	1.731726 × 10${}^{8}$
4	1020	409	81,474,905 $\times 10^{6}$	2.4499248 × 10${}^{8}$

## Data Availability

Not applicable.

## Abbreviations

The following abbreviations are used in this manuscript:

ACP	Algebra of Communicating Processes
AI	Artificial Intelligence
AIoT	Artificial Intelligence of Things
ANN	Artificial Neural Networks
ASIC	Application-specific integrated circuit
AV	Autonomous Vehicles
CL	Centralized Learning
CNN	Convolutional Neural Networks
DL	Deep Learning
DS	Data Science
EA	Evolutionary Algorithm
ECNN	Evolutionary Convolutional Neural Networks
FDT	Formal Description Techniques
FL	Federated Learning
G-IoT	Green Internet of Things
GPU	Graphical Processing Unit
HFCL	Hybrid Federated Centralized Learning
IIoT	Industrial Internet of Things
IoFT	Internet of Federated Things
IoT	Internet of Things
LAN	Local Area Network
LTL	Linear Temporal Logic
MEC	Multi-Access Edge Computing
MLP	Multi Layer Perceptron
MSC	Message Sequence Chart
MSG	Message
ML	Machine Learning
OSI	Open Systems Interconnection
Pub/Sub	Publisher/Subscriber
PS	Parameter Server
PROMELA	PROtocol/PROcess MEta LAnguage
RAN	Radio Access Network
ReLu	Rectified Linear Activation Function
RNN	Recurrent Neural Networks
SPIN	Simple Promela INterpreter
VANET	Vehicular Ad-Hoc Networks
WAN	Wide Area Network

## References

[1] Deng S., Zhao H., Fang W., Yin J., Dustdar S., Zomaya A.Y. (2020). Edge Intelligence: The Confluence of Edge Computing and Artificial Intelligence. IEEE Internet Things J..

[2] Hao C., Dotzel J., Xiong J., Benini L., Zhang Z., Chen D. (2021). Enabling Design Methodologies and Future Trends for Edge AI: Specialization and Codesign. IEEE Des. Test.

[3] Agarwal G.K., Magnusson M., Johanson A. (2021). Edge AI Driven Technology Advancements Paving Way Towards New Capabilities. Int. J. Innov. Technol. Manag..

[4] Quasim M.T. (2021). Resource Management and Task Scheduling for IoT using Mobile Edge Computing. Wirel. Pers. Commun..

[5] Rong G., Xu Y., Tong X., Fan H. (2021). An edge-cloud collaborative computing platform for building AIoT applications efficiently. J. Cloud Comput..

[6] Wang Q., Liu L., Zhang S., Lau F. (2021). On Massive IoT Connectivity with Temporally-Correlated User Activity. arXiv.

[7] Shafique M., Marchisio A., Putra R.V.W., Hanif M.A. (2021). Towards Energy-Efficient and Secure Edge AI: A Cross-Layer Framework. arXiv.

[8] Shahidinejad A., Farahbakhsh F., Ghobaei-Arani M., Malik M.H., Anwar T. (2021). Context-Aware Multi-User Offloading in Mobile Edge Computing: A Federated Learning-Based Approach. J. Grid Comput..

[9] Bibi R., Saeed Y., Zeb A., Ghazal T.M., Rahman T., Said R.A., Abbas S., Ahmad M., Khan M.A. (2021). Edge AI-Based Automated Detection and Classification of Road Anomalies in VANET Using Deep Learning. Comput. Intell. Neurosci..

[10] Artificial Intelligence Technology: AI Trends That Matter for Business. https://mobidev.biz/blog/future-artificial-intelligence-technology-ai-trends/.

[11] Low Code and No Code Platforms for AI and Computer Vision. https://viso.ai/computer-vision/low-code-ai-for-computer-vision/.

[12] Iyer C.K., Hou F., Wang H., Wang Y., Oh K., Ganguli S., Pandy P. (2021). Trinity: A No-Code AI platform for complex spatial datasets. arXiv.

[13] Top 12 ‘No-Code’ Machine Learning Platforms in 2021. https://analyticsindiamag.com/can-businesses-rely-entirely-on-no-code-low-code-platforms/.

[14] Using IoT for Smart Office Automation. https://mobidev.biz/blog/using-iot-for-smart-office-automation/.

[15] Zhu M., Sun Z., Chen T., Lee C. (2021). Low cost exoskeleton manipulator using bidirectional triboelectric sensors enhanced multiple degree of freedom sensory system. Nat. Commun..

[16] Silva M.C., da Silva J.C., Delabrida S., Bianchi A.G., Ribeiro S.P., Silva J.S., Oliveira R.A. (2021). Wearable Edge AI Applications for Ecological Environments. Sensors.

[17] Mohan H.M., Anitha S., Chai R., Ling S.H. (2021). Edge Artificial Intelligence: Real-Time Noninvasive Technique for Vital Signs of Myocardial Infarction Recognition Using Jetson Nano. Adv.-Hum.-Comput. Interact..

[18] Yacoub A., Hamri M.E., Frydman C. (2020). DEv-PROMELA: Modeling, verification, and validation of a video game by combining model-checking and simulation. SIMULATION—Trans. Soc. Model. Simul. Int..

[19] Ben-Ari M. (2008). Principles of the Spin Model Checker.

[20] Fokkink W. (2007). Introduction to Process Algebra.

[21] Iqbal I.M., Adzkiya D., Mukhlash I. (2017). Formal verification of automated teller machine systems using SPIN. Proc. AIP Conf..

[22] Gleirscher M., Marmsoler D. (2020). Formal Methods in Dependable Systems Engineering: A Survey of Professionals from Europe and North America. Empir. Softw. Eng..

[23] Smolinski M. (2017). Resolving Classical Concurrency Problems Using Outlier Detection. J. Appl. Comput. Sci..

[24] Roig P.J., Alcaraz S., Gilly K., Bernad C., Juiz C. (2022). Arithmetic Framework to Optimize Packet Forwarding among End Devices in Generic Edge Computing Environments. Sensors.

[25] Al-Fares M., Loukissas A., Vahdat A. (2008). A scalable, commodity data center network architecture. ACM SIGCOMM Comput. Commun. Rev..

[26] Umair M., Cheema M.A., Cheema O., Li H., Lu H. (2021). Impact of COVID-19 on IoT Adoption in Healthcare, Smart Homes, Smart Buildings, Smart Cities, Transportation and Industrial IoT. Sensors.

[27] Iggena T., Bin I.E., Fischer M., Tönjes R., Elsaleh T., Rezvani R., Pourshahrokhi N., Bischof S., Fernbach A., Xavier P.J. (2021). IoTCrawler: Challenges and Solutions for Searching the Internet of Things. Sensors.

[28] Ferreira C.M.S., Garrocho C.T.B., Oliveira R.A.R., Silva J.S., Cavalcanti C.F.M.D.C. (2021). IoT Registration and Authentication in Smart City Applications with Blockchain. Sensors.

[29] Internet of Things (IOT) Market—Growth, Trends, Covid-19 Impact, and Forecasts (2021–2026). https://www.mordorintelligence.com/industry-reports/internet-of-things-moving-towards-a-smarter-tomorrow-market-industry/.

[30] How Many IoT Devices Are There in 2021? [All You Need to Know]. https://techjury.net/blog/how-many-iot-devices-are-there/.

[31] IoT Trends to Drive Innovation for Business in 2021. https://mobidev.biz/blog/iot-technology-trends/.

[32] Yang C.T., Chen H.W., Chang E.J., Kristiani E., Nguyen K.L.P., Chang J.S. (2021). Current advances and future challenges of AIoT applications in particulate matters (PM) monitoring and control. J. Hazard. Mater..

[33] Wan W., Kubendran R., Schaefer C., Eryilmaz S.B., Zhang W., Wu D., Deiss S., Raina P., Qian H., Gao B. (2021). Edge AI without Compromise: Efficient, Versatile and Accurate Neurocomputing in Resistive Random-Access Memory. arXiv.

[34] Armgarth A., Pantzare S., Arven P., Lassnig R., Jinno H., Gabrielsson E.O., Kifle Y., Cherian D., Sjöström T.A., Berthou G. (2021). A digital nervous system aiming toward personalized IoT healthcare. Sci. Rep..

[35] Cheruvu S., Kumar A., Smith N., Wheeler D.M. (2020). Connectivity Technologies for IoT. Demystifying Internet of Things Security.

[36] Bauer M., Sánchez L., Song J.S. (2021). IoT-Enabled Smart Cities: Evolution and Outlook. Sensors.

[37] Pradhan B., Bhattacharyya S., Pal K. (2021). IoT-Based Applications in Healthcare Devices. Med. Internet Things (IoT) Devices.

[38] Vargas J., Alsweiss S., Toker O., Razdan R., Santos J. (2021). An Overview of Autonomous Vehicles Sensors and Their Vulnerability to Weather Conditions. Sensors.

[39] Dhirani L.L., Armstrong E., Newe T. (2021). Industrial IoT, Cyber Threats, and Standards Landscape: Evaluation and Roadmap. Sensors.

[40] Fraga-Lamas P., Lopes S.I., Fernández Caramés T.M. (2021). Green IoT and Edge AI as Key Technological Enablers for a Sustainable Digital Transition towards a Smart Circular Economy: An Industry 5.0 Use Case. Sensors.

[41] Kjorveziroski V., Filiposka S., Trajkovic V. (2021). IoT Serverless Computing at the Edge: Open Issues and Research Direction. Computers.

[42] Nguyen D.C., Ding M., Pathirana P.N., Seneviratne A., Li J., Poor H.V. (2021). Federated Learning for Internet of Things: A Comprehensive Survey. arXiv.

[43] Kontar R., Shi N., Yue X., Chung S., Byon E., Chowdhury M., Jin J., Kontar W., Masoud N., Nouiehed M. (2021). The Internet of Federated Things. IEEE Access.

[44] Famitafreshi G., Afaqui M.S., Melià-Seguí J. (2021). A Comprehensive Review on Energy Harvesting Integration in IoT Systems from MAC Layer Perspective: Challenges and Opportunities. Sensors.

[45] How Will Adopting an Edge Computing Strategy Benefit Organizations?. https://www.intelligentcio.com/apac/2021/08/23/how-will-adopting-an-edge-computing-strategy-benefit-organizations/.

[46] Next-Generation IoT and Edge Computing Strategy Forum Summary. https://digital-strategy.ec.europa.eu/en/library/next-generation-iot-and-edge-computing-strategy-forum-summary/.

[47] Mouha R.A. (2021). Internet of Things (IoT). J. Data Anal. Inf. Process..

[48] Bi Z., Jin Y., Maropoulos P., Zhang W.J., Wang L. (2021). Internet of things (IoT) and big data analytics (BDA) for digital manufacturing (DM). Int. J. Prod. Res..

[49] Mazón-Olivo B., Pan A. (2021). Internet of Things: State-of-the-art, Computing Paradigms and Reference Architectures. IEEE Lat. Am. Trans..

[50] Comini M., Gallardo M.M., Villanueva A. (2021). A denotational semantics for PROMELA addressing arbitrary jumps. arXiv.

[51] Krishnan R., Lalithambika V.R. (2020). Modeling and Validating Launch Vehicle Onboard Software Using the SPIN Model Checker. J. Aerosp. Inf. Syst..

[52] Shimakawa M., Iwasaki Y., Hagihara S., Yonezaki N. Discussion of LTL Subsets for Efficient Verification. Proceedings of the Workshop on Theory and Practice of Computation.

[53] Introduction to Promela. http://spinroot.com/spin/Man/Intro.html/.

[54] Zakarija I., Škopljanac-Mačina F. (2020). Automated simulation and verification of process models discovered by process mining. Autom. J. Control. Meas. Electron. Comput. Commun..

[55] Kulik T., Boudjadar J., Tran-Jorgesen P.W.V. Security Verification of Industrial Control Systems using Partial Model Checking. Proceedings of the 8th International Conference on Formal Methods in Software Engineering.

[56] Molero X., Juiz C., Rodeño M. (2004). Evaluación y Modelado del Rendimiento de los Sistemas Informáticos.

[57] Groote J.F., Mousavi M.R. (2014). Modeling and Analysis of Communicating Systems.

[58] Bergstra J.A., Middleburg C.A. (2021). Using Hoare Logic in a Process Algebra Setting. Fundam. Inform..

[59] Fokkink W. (2017). Modelling Distributed Systems.

[60] Bergstra J.A., Middleburg C.A. (2020). Process algebra with strategic interleaving. arXiv.

[61] Alcaraz S., Roig P.J., Gilly K., Filiposka S., Aknin N. Formal Algebraic Description of a Fog/IoT Computing Environment. Proceedings of the 24th International Conference Electronics.

